# Novel Toxin-Antitoxin Module SlvT-SlvA Regulates Megaplasmid Stability and Incites Solvent Tolerance in Pseudomonas putida S12

**DOI:** 10.1128/AEM.00686-20

**Published:** 2020-06-17

**Authors:** Hadiastri Kusumawardhani, David van Dijk, Rohola Hosseini, Johannes H. de Winde

**Affiliations:** a Institute of Biology Leiden, Leiden University, Leiden, The Netherlands; University of Tokyo

**Keywords:** genome engineering, RND efflux pump, toxin-antitoxin, megaplasmid, solvent tolerance, industrial biotechnology

## Abstract

Pseudomonas putida S12 is highly tolerant of organic solvents in saturating concentrations, rendering this microorganism suitable for the industrial production of various aromatic compounds. Previous studies revealed that P. putida S12 contains the single-copy 583-kbp megaplasmid pTTS12. pTTS12 carries several important operons and gene clusters facilitating P. putida S12 survival and growth in the presence of toxic compounds or other environmental stresses. We wished to revisit and further scrutinize the role of pTTS12 in conferring solvent tolerance. To this end, we cured the megaplasmid from P. putida S12 and conclusively confirmed that the SrpABC efflux pump is the major determinant of solvent tolerance on the megaplasmid pTTS12. In addition, we identified a novel toxin-antitoxin module (proposed gene names *slvT* and *slvA,* respectively) encoded on pTTS12 which contributes to the solvent tolerance phenotype and is important for conferring stability to the megaplasmid. Chromosomal introduction of the *srp* operon in combination with the *slv*AT gene pair created a solvent tolerance phenotype in non-solvent-tolerant strains, such as P. putida KT2440, Escherichia coli TG1, and E. coli BL21(DE3).

**IMPORTANCE** Sustainable alternatives for high-value chemicals can be achieved by using renewable feedstocks in bacterial biocatalysis. However, during the bioproduction of such chemicals and biopolymers, aromatic compounds that function as products, substrates, or intermediates in the production process may exert toxicity to microbial host cells and limit the production yield. Therefore, solvent tolerance is a highly preferable trait for microbial hosts in the biobased production of aromatic chemicals and biopolymers. In this study, we revisit the essential role of megaplasmid pTTS12 from solvent-tolerant Pseudomonas putida S12 for molecular adaptation to an organic solvent. In addition to the solvent extrusion pump (SrpABC), we identified a novel toxin-antitoxin module (SlvAT) which contributes to short-term tolerance in moderate solvent concentrations, as well as to the stability of pTTS12. These two gene clusters were successfully expressed in non-solvent-tolerant strains of P. putida and Escherichia coli strains to confer and enhance solvent tolerance.

## Abstract (precis)

Sustainable alternatives for high-value chemicals can be achieved by using renewable feedstocks in bacterial biocatalysis. However, during the bioproduction of such chemicals and biopolymers, aromatic compounds that function as products, substrates, or intermediates in the production process may exert toxicity to microbial host cells and limit the production yield. Therefore, solvent tolerance is a highly preferable trait for microbial hosts in the biobased production of aromatic chemicals and biopolymers. In this study, we revisit the essential role of megaplasmid pTTS12 from solvent-tolerant Pseudomonas putida S12 for molecular adaptation to an organic solvent. In addition to the solvent extrusion pump (SrpABC), we identified a novel toxin-antitoxin module (SlvAT) which contributes to short-term tolerance in moderate solvent concentrations, as well as to the stability of pTTS12. These two gene clusters were successfully expressed in non-solvent-tolerant strains of P. putida and Escherichia coli strains to confer and enhance solvent tolerance.

## INTRODUCTION

One of the main challenges in the production of aromatic compounds is chemical stress caused by the added substrates, pathway intermediates, or products. These chemicals, often exhibiting characteristics of organic solvents, are toxic to microbial hosts and may negatively impact product yields. They adhere to the cell membranes, alter membrane permeability, and cause membrane damage (1, 2). Pseudomonas putida S12 exhibits exceptional solvent tolerance characteristics, enabling this strain to withstand toxic organic solvents in saturating concentrations (3, 4). Consequently, a growing list of valuable compounds has successfully been produced using P. putida S12 as a biocatalyst by exploiting its solvent tolerance (5–9).

Following the completion of its full-genome sequence and subsequent transcriptome and proteome analyses, several genes have been identified that may play important roles in controlling and maintaining solvent tolerance of P. putida S12 (10–12). As previously reported, an important solvent tolerance trait of P. putida S12 is conferred through the resistance-nodulation-division (RND)-family efflux pump SrpABC, which actively removes organic solvent molecules from the cells (13, 14). Initial attempts to heterologously express the SrpABC efflux pump in Escherichia coli enabled the instigation of solvent tolerance and production of 1-naphthol (15, 16). Importantly, the SrpABC efflux pump is encoded on the megaplasmid pTTS12 of P. putida S12 (12).

The 583-kbp megaplasmid pTTS12 is a stable single-copy plasmid specific to P. putida S12 (12). It harbors several important operons and gene clusters enabling P. putida S12 to tolerate, resist, and survive the presence of various toxic compounds or otherwise harsh environmental conditions. Several examples include the presence of a complete styrene degradation pathway gene cluster, the RND efflux pump specialized for organic solvents (SrpABC), and several gene clusters conferring heavy metal resistance (12, 17, 18). In addition, through analysis using TADB2.0, a toxin-antitoxin database (19, 20), pTTS12 is predicted to contain three toxin-antitoxin modules. Toxin-antitoxin modules recently have been recognized as important determinants of resistance toward various stress conditions, like nutritional stress and exposure to sublethal concentration of chemical stressors (21, 22). Toxin-antitoxin modules identified in pTTS12 consist of an uncharacterized RPPX_26255-RPPX_26260 system and two identical copies of a VapBC system (23). RPPX_26255 and RPPX_26260 belong to a newly characterized type II toxin-antitoxin pair, COG5654-COG5642. While toxin-antitoxin systems are known to preserve plasmid stability through postsegregational killing of plasmid-free daughter cells (24), RPPX_26255-RPPX_26260 was also previously shown to be upregulated during organic solvent exposure, suggesting its role in solvent tolerance (11).

In the manuscript, we further address the role of pTTS12 in conferring solvent tolerance of P. putida S12. Curing pTTS12 from its host strain might cause a reduction in solvent tolerance, while complementation of the *srp* operon into the cured strain may fully or partially restore solvent tolerance. Furthermore, we wished to identify additional genes or gene clusters on pTTS12 and putative mechanisms that might also play a role in conferring solvent tolerance to P. putida and non-solvent-tolerant E. coli.

## RESULTS

### Megaplasmid pTTS12 is essential for solvent tolerance in P. putida S12.

**FIG 1:**
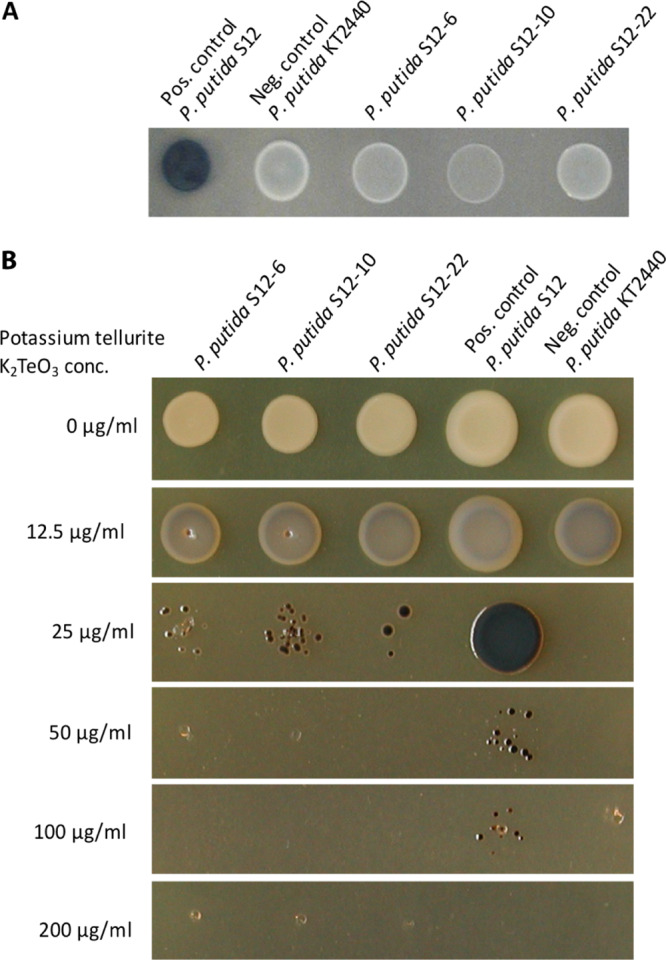
Curing of the megaplasmid pTTS12 from P. putida S12. (A) Activity of styrene monooxygenase (SMO) and styrene oxide isomerase (SOI) for indigo formation from indole in P. putida strains. Enzyme activity was lost in the megaplasmid-cured genotype S12 ΔpTTS12 (white colonies). Indole (100 mg liter^−1^) was supplemented in M9 minimum medium. (B) K_2_TeO_3_ resistance of P. putida genotypes on lysogeny broth (LB) agar. Tellurite resistance was reduced in the megaplasmid-cured genotype S12 ΔpTTS12 (MIC, 50 mg liter^−1^).

To further analyze the role of the megaplasmid of P. putida S12 in solvent tolerance, pTTS12 was removed from P. putida S12 using mitomycin C. This method was selected due to its reported effectiveness in removing plasmids from *Pseudomonas* sp. (25), although previous attempts regarded plasmids that were significantly smaller in size than pTTS12 (26). After treatment with mitomycin C (10 to 50 mg liter^−1^), liquid cultures were plated on M9 minimal medium supplemented with indole to select for plasmid-cured colonies. Megaplasmid pTTS12 encodes two key enzymes, namely, styrene monooxygenase (SMO) and styrene oxide isomerase (SOI) that are responsible for the formation of indigo coloration from indole. This conversion results in indigo coloration in spot assays for wild-type P. putida S12, whereas white colonies are formed in the absence of megaplasmid pTTS12. With the removal of pTTS12, a loss of indigo coloration and, hence, of indigo conversion was observed in all three plasmid-cured strains and the negative control P. putida KT2440 (Fig. 1A).

With a mitomycin C concentration of 30 mg liter^−1^, 2.4% (3 out of 122) of the obtained colonies appeared to be completely cured of the megaplasmid, underscoring the high genetic stability of the plasmid. No colonies survived the addition of 40 and 50 mg liter^−1^ of mitomycin C, whereas all the colonies that survived the addition of 10 and 20 mg liter^−1^ of mitomycin C retained the megaplasmid. Three independent colonies cured from the megaplasmid were isolated as P. putida S12-6, P. putida S12-10, and P. putida S12-22. The complete loss of the megaplasmid was further confirmed by phenotypic analysis (Fig. 1) and by full-genome sequencing. Several operons involved in heavy metal resistance were previously reported in the pTTS12 (12). The *terZABCD* operon contributes to tellurite resistance in wild-type P. putida S12 with MICs as high as 200 mg liter^−1^ (Fig. 1B). In the megaplasmid-cured strains, a severe reduction of tellurite resistance was observed, decreasing the potassium tellurite MIC to 50 mg liter^−1^ (Fig. 1B).

Genomic DNA sequencing confirmed a complete loss of pTTS12 from P. putida genotypes S12-6, S12-10, and S12-22 without any plasmid-derived fragment being inserted within the chromosome, and genomic alterations by mitomycin C treatment were minimal. Complementation of pTTS12 into the plasmid-cured P. putida S12 genotypes restored the indole-indigo transformation and high tellurite resistance to a similar level as the wild-type strain (see Fig. S1 in the supplemental material). Repeated megaplasmid curing experiments indicated that P. putida S12 can survive the addition of 30 mg liter^−1^ mitomycin C with the frequency of (2.48 ± 0.58) × 10^−8^. Among these survivors, only 2% of the colony population lost the megaplasmid, confirming the genetic stability of pTTS12. In addition, attempts to cure the plasmid by introducing double-strand breaks as described by Wynands and colleagues (27) were not successful due to the pTTS12 stability.

**FIG 2:**
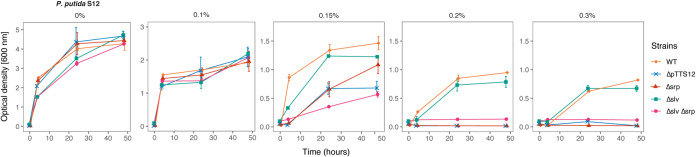
Megaplasmid pTTS12 determines the solvent tolerance trait of P. putida S12. Solvent tolerance analysis was performed on wild-type P. putida S12, P. putida S12 ΔpTTS12 (genotypes S12-6, S12-10, and S12-22), P. putida S12 Δsrp, P. putida S12 Δslv, and P. putida S12 Δsrp Δslv growing in liquid LB media with 0%, 0.10%, 0.15%, 0.20%, and 0.30% (vol/vol) toluene. The removal of the megaplasmid pTTS12 clearly caused a significant reduction in the solvent tolerance of P. putida S12 ΔpTTS12. Deletion of srpABC (Δsrp), RPPX_26255-RPPX_26260 (Δ*slv*), and the combination of these gene clusters (Δ*srp* Δ*slv*) resulted in a lower solvent tolerance. This figure displays the means of three biological replicates, and error bars indicate standard deviation. The ranges of the *y* axes are different in the first panel (0 to 5), second panel (0 to 3), and third to fifth panels (0 to 1.5).

Growth comparison in solid and liquid culture in the presence of toluene was performed to analyze the effect of megaplasmid curing in constituting the solvent tolerance trait of P. putida S12. In contrast with wild-type P. putida S12, the plasmid-cured genotypes were unable to grow under toluene atmosphere conditions (data not shown). In liquid LB medium, plasmid-cured P. putida S12 genotypes were able to tolerate 0.15% (vol/vol) toluene, whereas the wild-type P. putida S12 could grow in the presence of 0.30% (vol/vol) toluene (Fig. 2). In the megaplasmid-complemented P. putida S12-C genotypes, solvent tolerance was restored to the wild-type level (Fig. S1D). Hence, the absence of megaplasmid pTTS12 caused a significant reduction of solvent tolerance in P. putida S12.

### The SrpABC efflux pump and gene pair RPPX_26255-RPPX_26260 are the main constituents of solvent tolerance encoded on pTTS12.

The significant reduction of solvent tolerance in plasmid-cured P. putida S12 underscored the important role of megaplasmid pTTS12 in solvent tolerance. Besides encoding the efflux pump SrpABC enabling efficient intermembrane solvent removal (12, 13), pTTS12 carries more than 600 genes and, hence, may contain additional genes involved in solvent tolerance. Two adjacent hypothetical genes, RPPX_26255 and RPPX_26260, were previously reported to be upregulated in a transcriptomic study as a short-term response to toluene addition (11). We propose to name the RPPX_26255-RPPX_26260 gene pair as “*slv*” due to its elevated expression in the presence of solvent. In a first attempt to identify additional potential solvent tolerance regions of pTTS12, we deleted the *srp*ABC genes (Δ*srp*), RPPX_26255-RPPX_26260 genes (Δ*slv*), and the combination of both gene clusters (Δ*srp* Δ*slv*) from pTTS12 in wild-type P. putida S12.

All strains were compared for growth under increasing toluene concentrations in liquid LB medium (Fig. 2). In the presence of low concentrations of toluene (0.10% [vol/vol]), all genotypes showed similar growth. With the addition of 0.15% (vol/vol) toluene, S12 Δ*slv*, S12 Δ*srp*, and S12 Δ*srp* Δ*slv* exhibited slower growth and reached a lower optical density at 600 nm (OD_600_) than the wild-type S12 strain. S12 Δ*slv* and S12 Δ*srp* achieved a higher OD_600_ in batch growth than S12 ΔpTTS12 and S12 Δ*srp* Δ*slv* due to the presence of the SrpABC efflux pump or RPPX_26255-RPPX_26260 gene pair.

Interestingly, S12 Δ*srp* Δ*slv* (still containing pTTS12) exhibited diminished growth compared with S12 ΔpTTS12. This may be an indication of megaplasmid burden in the absence of essential genes for solvent tolerance. With 0.20% and 0.30% (vol/vol) toluene added to the medium, S12 Δ*srp*, S12 Δ*srp* Δ*slv*, and S12 ΔpTTS12 were unable to grow, while wild-type S12 and S12 Δ*slv* were able to grow, although S12 Δ*slv* reached a lower OD_600_. Taken together, these results demonstrate an important role for both the SrpABC efflux pump and the *slv* gene pair in conferring solvent tolerance. We chose P. putida S12-6 for further experiments representing megaplasmid-cured P. putida S12.

### Solvent tolerance can be exerted by ectopic expression of the SrpABC efflux pump and *slv* gene pair in Gram-negative bacteria.

The functionality of the *srp* operon and *slv* gene pair was explored in the model Gram-negative non-solvent-tolerant strains P. putida KT2440, E. coli TG1, and E. coli BL21(DE3). We complemented *srpRSABC* (*srp* operon), *slv* gene pair, and a combination of both gene clusters into P. putida S12-6, P. putida KT2440, E. coli TG1, and E. coli BL21(DE3) using mini-Tn7 transposition. These strains were chosen due to their common application as model industrial strains while lacking solvent tolerance. P. putida KT2440 is another robust microbial host for metabolic engineering due to its adaptation toward physicochemical stresses; however, contrary to P. putida S12, this strain is not solvent tolerant (28). E. coli BL21(DE3), derived from strain B, is the common E. coli lab strain optimized for protein production due to its lacking Lon and OmpT proteases and encoding T7 RNA polymerase (29). E. coli TG1 was previously reported to successfully produce 1-naphthol with the expression of SrpABC (15, 16), and therefore, this strain was included in this study as a comparison.

**FIG 3:**
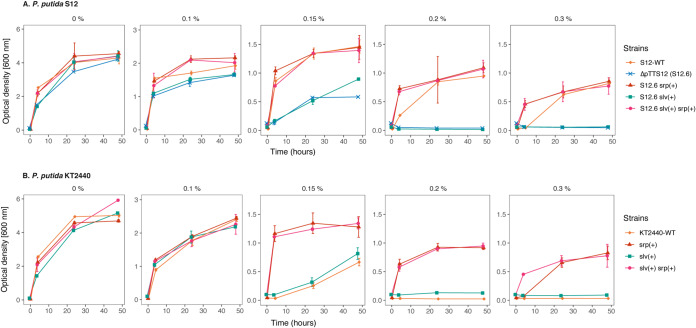
Chromosomal introduction of *srp* and *slv* gene clusters increased solvent tolerance in P. putida genotypes. Solvent tolerance analysis of the genotypes with chromosomal introduction of *srp* operon (*srpRSABC*), *slv* gene pair (RPPX_26255-RPPX_26260), and the combination of these gene clusters into P. putida S12 ΔpTTS12 (represented by strain S12-6) (A) and wild-type P. putida KT2440 (B) in liquid LB with 0%, 0.10%, 0.15%, 0.20%, and 0.30% (vol/vol) of toluene. Wild-type P. putida S12 was taken as a solvent-tolerant control strain. This figure displays the mean of three independent replicates, and error bars indicate standard deviation. The ranges of *y* axes are different in the first panel (0 to 6), second panel (0 to 3), and third to fifth panels (0 to 1.5).

The chromosomal introduction of *slv* into S12-6 and KT2440 improved the growth of the resulting strains at 0.15% (vol/vol) toluene compared with S12-6 and KT2440 (Fig. 3). The introduction of *srp* or a combination of *slv* and *srp* enables S12-6 and KT2440 to grow in the presence of 0.30% (vol/vol) toluene. In KT2440, the introduction of both *slv* and *srp* resulted in a faster growth in the presence of 0.30% (vol/vol) toluene than the addition of only *srp* (Fig. 3B). Interestingly, the growth of S12-6 *srp*,*slv* and S12.6 *srp* is better than wild-type S12 (Fig. 3A). The observed faster growth of S12-6 *srp*,*slv* and S12.6 *srp* may be due to more efficient growth in the presence of toluene, supported by a chromosomally introduced *srp* operon, than its original megaplasmid localization. Indeed, replication of this large megaplasmid is likely to require additional maintenance energy. To corroborate this, we complemented the megaplasmid pTTS12 lacking the solvent pump (Tc^r^::*srpABC*) into P. putida S12-6 *srp,* resulting in P. putida S12-9. Indeed, P. putida S12-9 showed further reduced growth in the presence of 0.20 and 0.30% toluene (see Fig. S2 in the supplemental material), indicating the metabolic burden of carrying the megaplasmid. We conclude that the SrpABC efflux pump can be regarded as the major contributor to solvent tolerance from pTTS12. The *slv* gene pair appears to promote the tolerance of P. putida S12 at least under moderate solvent concentrations.

**FIG 4:**
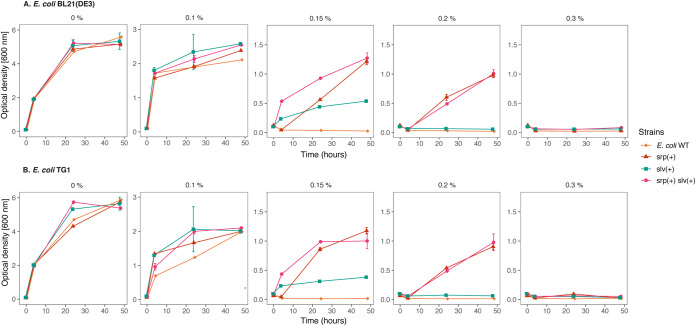
Chromosomal introduction of *srp* and *slv* gene clusters increased solvent tolerance in E. coli strains. Solvent tolerance analysis of the strains with chromosomal introduction of *srp* operon (*srpRSABC*), *slv* gene pair (RPPX_26255-RPPX_26260), and the combination of these gene clusters into E. coli BL21(DE3) (A) and E. coli TG1 (B) in liquid LB with 0%, 0.10%, 0.15%, 0.20%, and 0.30% (vol/vol) of toluene. This figure displays the mean of three independent replicates, and error bars indicate standard deviation. The ranges of *y* axes are different in the first panel (0 to 6), second panel (0 to 3), and third to fifth panels (0 to 1.5).

The intrinsic solvent tolerance of E. coli strains was observed to be clearly lower than that of P. putida (Fig. 4). The wild-type E. coli strains were able to withstand a maximum 0.10% (vol/vol) toluene, whereas plasmid-cured P. putida S12-6 and P. putida KT2440 were able to grow in the presence of 0.15% (vol/vol) toluene. With the introduction of *slv* and *srp* in both E. coli strains, solvent tolerance was increased up to 0.15% and 0.20% (vol/vol) toluene, respectively (Fig. 4). A combination of *slv* and *srp* also increased tolerance to 0.20% (vol/vol) toluene, while showing a better growth than the chromosomal introduction of only *srp*. However, none of these strains were able to grow in the presence of 0.30% (vol/vol) toluene.

qPCR analysis of SrpABC expression (see Table S1 in the supplemental material) in P. putida S12, P. putida KT2440, E. coli TG1, and E. coli BL21(DE3) confirmed that *srp*A, *srp*B, and *srp*C were expressed at basal levels in all strains. In the presence of 0.10% toluene, the expression of *srp*A, *srp*B, and *srp*C was clearly upregulated in all strains. Thus, the lower solvent tolerance conferred by introducing the SrpABC efflux pump in E. coli strains was not due to the lower expression of the *srp* genes. An analysis of the codon adaptation index (CAI) (http://ppuigbo.me/programs/CAIcal/) (30) showed that for both the P. putida and E. coli strains, the CAI values of the *srp* operon are suboptimal, clearly below 0.8 to 1.0 (see Table S2 in the supplemental material). Interestingly, the CAI values were higher for E. coli (0.664) than for P. putida (0.465), predicting a better protein translation efficiency of the *srp* operon in E. coli. Hence, reduced translation efficiency is not likely to be the cause of lower performance of the *srp* operon in E. coli strains for generating solvent tolerance. Overall, our results indicate that, in addition to the solvent efflux pump, P. putida S12 and P. putida KT2440 are intrinsically more robust than E. coli TG1 and E. coli BL21(DE3) in the presence of toluene.

### The *slv* gene pair constitutes a novel toxin-antitoxin system.

BLASTp analysis was initiated to further characterize RPPX_26255 and RPPX_26260. This analysis indicated that RPPX_26260 and RPPX_26255 likely represent a novel toxin-antitoxin (TA) system. Through a database search on TADB2.0 (19, 20), we found that RPPX_26260 is a toxin of the COG5654 family and typically carries an RES domain-containing protein, which has a conserved arginine (R)-glutamine (E)-serine (S) motif providing a putative active site; and RPPX_26255 is an antitoxin of the COG5642 family. Based on its involvement in solvent tolerance, we propose naming the toxin-encoding RPPX_26260 as *slvT* and the antitoxin-encoding RPPX_26255 as *slvA*.

**FIG 5:**
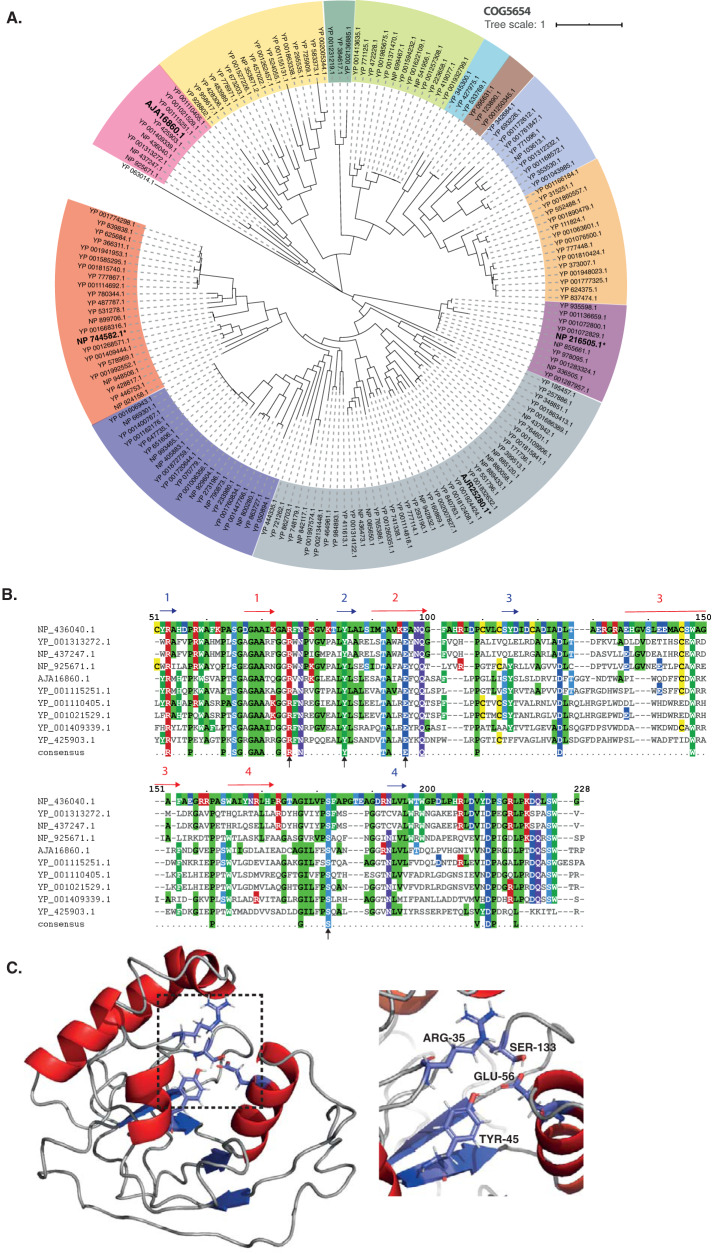
Bioinformatics analysis of SlvT as a member of the COG5654 toxin family. (A) Phylogenetic tree (neighbor-joining tree with 100 bootstraps) of COG5654 family toxin from reference sequences identified by Makarova and colleagues (31). Different colors correspond to the different toxin-antitoxin module clades. Asterisks (*) and bold text indicate the characterized toxin proteins, namely, ParT from *Sphingobium* sp. YBL2 (GenBank accession no. AJR25280.1), PP_2434 from P. putida KT2440 (NP_744582.1), MbcT from Mycobacterium tuberculosis H37Rv (NP_216505.1), and SlvT from P. putida S12 (AJA16860.1). (B) Multiple sequence alignment of the COG5654 toxin SlvT from P. putida S12 with several putative COG5654 family toxin proteins which belong in the same clade. Putative active site residues are indicated by black arrows. (C) Protein structure modeling of SlvT using the I-TASSER server (35), which exhibits high structural similarity with MbcT from Mycobacterium tuberculosis H37Rv. Shown are the close ups of putative active sites of the SlvT toxin (Arg-35, Tyr-45, Glu-56, and Ser-133).

**FIG 6:**
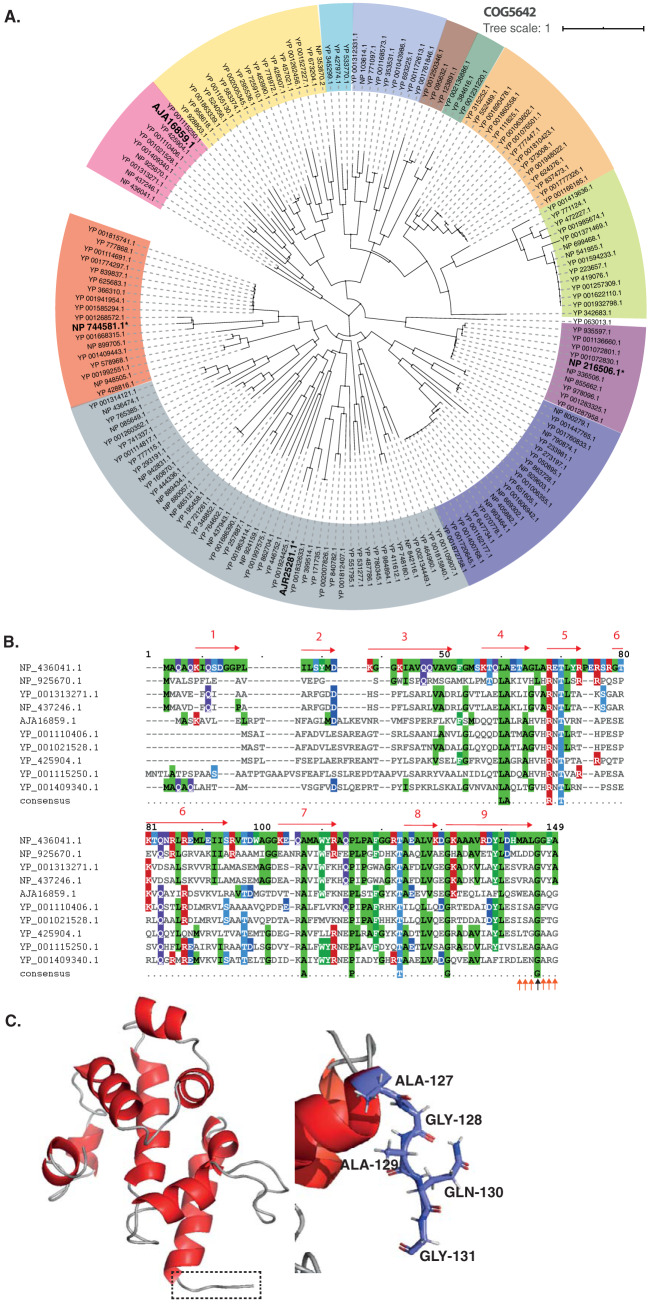
Bioinformatics analysis of SlvA as a member of the COG5642 toxin family. (A) Phylogenetic tree (neighbor-joining tree with 100 bootstraps) of the COG5642 family toxin from reference sequences identified by Makarova and colleagues (31). Different colors correspond to the different toxin-antitoxin module clades. Asterisks (*) and bold text indicate the characterized toxin proteins, namely, ParS from *Sphingobium* sp. YBL2 (GenBank accession no. AJR25281.1), PP_2433 from P. putida KT2440 (NP_744581.1), MbcA from Mycobacterium tuberculosis H37Rv (NP_216506.1), and SlvA from P. putida S12 (AJA16859.1). (B) Multiple sequence alignment of the COG5654 toxin SlvA from P. putida S12 with several putative COG5642 family toxin proteins which belong in the same clade. Putative active site residues are indicated by orange and black arrows. (C) Protein structure modeling of SlvA using the I-TASSER server (35), which exhibits high structural similarity with MbcA from Mycobacterium tuberculosis H37Rv. Shown are the close ups of the antitoxin putative C-terminal binding site to block the SlvT toxin active site (Ala-127, Gly-128, Ala-129, Gln-130, and Gly-131).

Makarova and colleagues identified putative toxin-antitoxin pairs through genome mining of reference sequences in the NCBI database (31). They identified 169 pairs of the COG5654-COG5642 TA system from the reference sequences. Here, we constructed a phylogenetic tree of the COG5654-COG5642 TA system, including SlvA (GenBank accession no. AJA16859.1) and SlvT (AJA16860.1), as shown in Fig. 5A and 6A. SlvA and SlvT cluster with other plasmid-borne toxin-antitoxin from Burkholderia vietnamensis G4, Methylibium petroleiphilum PM1, Rhodospirillum rubrum ATCC 11170, Xanthobacter autotrophicus Py2, Sinorhizobium meliloti 1021, Sinorhizobium medicae WSM419, and Gloeobacter violaceus PCC7421. Multiple alignments of SlvAT against the COG5654-COG5642 TA system are shown in Fig. 5B and 6B.

Of the 169 TA pairs of the COG5654-COG5642 TA system, three TA pairs have recently been characterized, namely, ParST from *Sphingobium* sp. YBL2 (GenBank accession no. AJR25281.1 and AJR25280.1) (32), PP_2433-PP_2434 from P. putida KT2440 (NP_744581.1 and NP_744582.1) (33), and MbcAT from Mycobacterium tuberculosis H37Rv (NP_216506.1 and NP_216505.1) (34), as indicated by bold text and asterisks in Fig. 5A and 6A. A 3D-model prediction of the SlvT and SlvA proteins using the I-TASSER suite for protein structure and function prediction (35) indicated that SlvT and SlvA showed the highest structural similarity to the MbcAT system from Mycobacterium tuberculosis (Fig. 5C and 6C), which is reported to be expressed during stress conditions (34). Amino acid conservation between SlvAT and these few characterized toxin-antitoxin pairs is relatively low, as they do not belong to the same clade (Fig. 5A and 6A). However, 100% conservation is clearly observed on the putative active side residues, namely, arginine (R) 35, tyrosine (Y) 45, and glutamine (E) 56, and only 75% consensus is shown on serine (S) 133 residue (see Fig. S3 in the supplemental material).

According to the model with the highest TM score, SlvT is predicted to consist of four beta sheets and four alpha helices. As such, SlvT shows structural similarity with diphtheria toxin which functions as an ADP-ribosyl transferase enzyme. Diphtheria toxin can degrade NAD^+^ into nicotinamide and ADP ribose (36). A similar function was recently identified for COG5654 family toxins from P. putida KT2440, M. tuberculosis, and *Sphingobium* sp. (32–34).

### *slvT* toxin causes cell growth arrest by depleting cellular NAD^+^.

**FIG 7:**
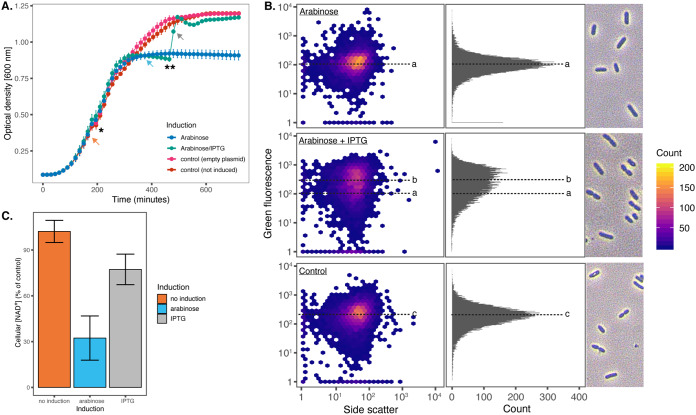
Heterologous expression of SlvAT in E. coli BL21(DE3). (A) Growth curves of E. coli BL21(DE3) harboring pBAD18-slvT and pUK21-slvA, showing growth reduction after the induction of toxin by a total concentration of 0.8% arabinose (*) and growth restoration after antitoxin induction by a total concentration of 2 mM IPTG (**). Samples were taken at the time points indicated by colored arrows for cellular NAD^+^ measurement. (B) Flow cytometry analysis of DNA content and cell morphology visualization on E. coli BL21(DE3) during *slvT* and *slvAT* expression. Median value of green fluorescence representing DNA content during *slvT* expression (118.202), *slvAT* expression (236.056), and control (208.406) are indicated by a, b, and c, respectively. Samples were taken at the time point indicated by the gray arrow in A. (C) Cellular NAD^+^ measurements during the expression of the toxin-antitoxin module. Induction of toxin SlvT caused a reduction in cellular NAD^+^ levels to 32.32% (± 14.47%) of the control strain, while the expression of SlvA restored the cellular NAD^+^ level to 77.27% (± 9.97%) of the control strain.

To prove that *slvAT* presents a pair of toxin and antitoxin, *slvA* and *slvT* were cloned separately in pUK21 (lac-inducible promoter) and pBAD18 (ara-inducible promoter), respectively. The two constructs were cloned into E. coli BL21(DE3). The growth of the resulting strains was monitored during conditional expression of the *slvA* and *slvT* genes (Fig. 7A). At the mid-log growth phase, a final concentration of 0.8% arabinose was added to the culture (*), inducing expression of *slvT*. After 2 h of induction, growth of this strain ceased, while the uninduced control culture continued to grow. Upon addition of 2 mM isopropyl-β-D-thiogalactopyranoside (IPTG) (**), growth of the *slvT-*induced culture was immediately restored, reaching a similar OD_600_ as the uninduced culture.

Bacterial cell division was further studied by flow cytometer analyses during the expression of *slvT* and *slvA*. After approximately 6 h of growth (indicated by gray arrow in Fig. 7A), samples were taken from control, arabinose, and arabinose + IPTG-induced liquid culture. Cell morphology was analyzed by light microscopy, and the DNA content of the individual cells in the culture was measured using a flow cytometer with SYBR green II staining (Fig. 7B). Indeed, an absence of dividing cells and lower DNA content were observed during the induction of only *slvT* toxin with arabinose (Fig. 7B). Subsequent addition of IPTG to induce *slvA* expression was shown to restore cell division and to an upshift of DNA content similar to that of control strain (Fig. 7B). While the expression of *slvT* was not observed to be lethal to the bacterial strain, this experiment showed that the expression of the *slvT* toxin stalled DNA replication and, subsequently, cell division. The induction of *slvA* subsequently restored bacterial DNA replication and cell division.

To corroborate a putative target of SlvT, concentrations of NAD^+^ were measured during the induction experiment (Fig. 7C). Before the addition of arabinose to induce *slvT* (orange arrow on Fig. 7A), NAD^+^ was measured and compared to the strain harboring empty pUK21 and pBAD18 (Fig. 7B). On average, at this time point, the NAD^+^ level is similar between the *slvAT*-bearing strain and the control strain. NAD^+^ was measured again after arabinose induction when the growth of the induced strain has diminished (blue arrow on Fig. 7A). At this time point, the measured NAD^+^ was 32% (± 14.47%) of the control strain. After the induction of *slvA*, NAD^+^ was immediately restored to a level of 77% (± 9.97%) compared with the control strain. Thus, the induction of *slvT* caused a depletion of NAD^+^, while induction of *slvA* immediately increased the NAD^+^ level, indicating that *slvAT* is a pair of toxin-antitoxin which controls its toxicity through NAD^+^ depletion.

### *slvAT* regulates megaplasmid pTTS12 stability.

In addition to its role in solvent tolerance, localization of the *slvAT* pair on megaplasmid pTTS12 may have an implications for plasmid stability. pTTS12 is a very stable megaplasmid that cannot be spontaneously cured from P. putida S12 and cannot be removed by introducing double-strand breaks (see above). We deleted *slvT* and *slvAT* from the megaplasmid to study their impact on pTTS12 stability. With the deletion of *slvT* and *slvAT*, the survival rate during treatment with mitomycin C improved significantly, reaching (1.01 ± 0.17) × 10^−4^ and (1.25 ± 0.81) × 10^−4^, respectively, while the wild-type S12 had a survival rate of (2.48 ± 0.58) × 10^−8^.

We determined the curing rate of pTTS12 from the surviving colonies. In wild-type S12, the curing rate was 2% (see also above), while in Δ*slvT* and Δ*slvAT* mutants, the curing rate increased to 41.3% (± 4.1%) and 79.3% (± 10%), respectively, underscoring an important role for *slvAT* in megaplasmid stability. We attempted to cure the megaplasmid from the colonies by introducing a double-strand break (DSB), as previously described on Pseudomonas taiwanensis VLB120 (27, 37). This indeed was not possible in wild-type S12 and Δ*slvT* strains; however, the Δ*slvAT* mutant now showed plasmid curing by a DSB, resulting in a curing rate of 34.3% (± 16.4%).

**FIG 8:**
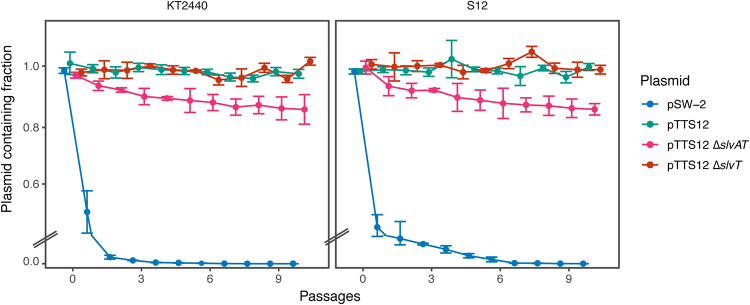
SlvAT is important for pTTS12 maintenance in P. putida. pTTS12 (variant with Km^r^) maintenance in P. putida S12 and P. putida KT2440 growing in LB liquid medium without antibiotic selection for 10 passages (approximately 10 generations per passage). pSW-2 was taken as a negative control for plasmid stability in P. putida. This experiment was performed with three biological replicates, and error bars represent standard deviation.

Since Δ*slvT* and Δ*slvAT* may compromise megaplasmid stability, we performed megaplasmid stability tests by growing S12 and KT2440 harboring pSW-2 (negative control) (37), pTTS12 (positive control), pTTS12 Δ*slvT*, and pTTS12 Δ*slvAT* in LB medium with 10 passages (± 10 generations/passage step) as shown in Fig. 8. Both KT2440 and S12 easily lost the negative-control plasmid pSW-2 (Fig. 8). Plasmid pTTS12 was not lost during this test, confirming that pTTS12 is indeed a stable plasmid. Furthermore, the Δ*slvT* genotypes also did not show a loss of the megaplasmid. Interestingly, the Δ*slvAT* genotypes spontaneously lost the megaplasmid, confirming that the *slvAT* module is not only important to promote solvent tolerance but also determines megaplasmid stability in P. putida S12 and KT2440.

## DISCUSSION

### Revisiting the role of pTTS12 and the SrpABC efflux pump in solvent tolerance.

**FIG 9:**
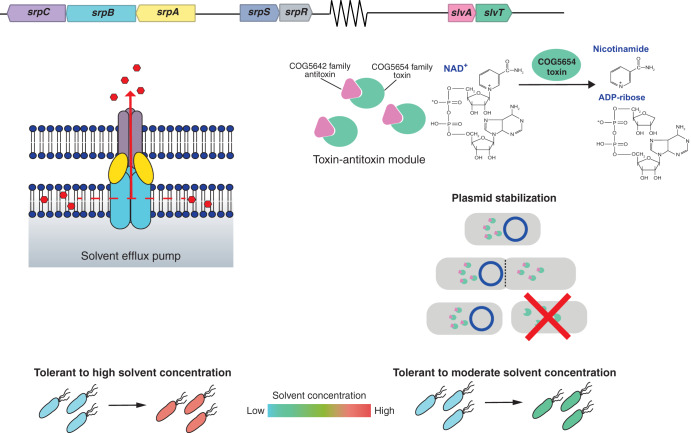
Schematic representation of the gene clusters involved in solvent tolerance from megaplasmid pTTS12. The SrpABC efflux pump is the major contributor to the solvent tolerance trait from the megaplasmid pTTS12. This efflux pump is able to efficiently extrude solvents from the membrane lipid bilayer. A COG5654-COG5642 family toxin-antitoxin module (SlvT and SlvA, respectively) promoted the growth of P. putida S12 in the presence of a moderate solvent concentration and stabilized the pTTS12 plasmid. In the absence of SlvA, SlvT causes toxicity by conferring cellular NAD^+^ depletion and, subsequently, halts DNA replication and cell division.

In this study, we conclusively confirm the role of the SrpABC efflux pump carried on pTTS12 and identify a novel toxin-antitoxin module playing an additional role in conveying solvent tolerance to P. putida S12 (Fig. 9). Notably, megaplasmids may cause a metabolic burden to their host strains, and they can be a source of genetic instability (11). Our results show that, indeed, pTTS12 imposed a metabolic burden in the presence of an organic solvent (Fig. S2). This plasmid is very large and contains many genes that are not related to solvent tolerance. Hence, it may be interesting for biotechnological purposes to reduce the plasmid size and, consequently, the metabolic burden. In addition, a streamlined and minimal genome size is desirable for reducing host interference and genome complexity (12, 13).

We investigated the heterologous expression of the SrpABC efflux pump in strains of both P. putida and E. coli, which successfully enhanced their solvent tolerance in these strains (Fig. 3 and 4). Previous reports on the implementation of SrpABC in whole-cell biocatalysis successfully increased the production of 1-naphthol in E. coli TG1 (15, 16). Production was still higher using P. putida S12, as this strain could better cope with substrate (naphthalene) toxicity, while both P. putida S12 and E. coli TG1 showed similar tolerance to the product 1-naphthol (16). In our experiments, the E. coli strains clearly showed a smaller increase in toluene tolerance than the P. putida strains, although *srpABC* was expressed at a basal level and upregulated in the presence of 0.10% (vol/vol). These results indicate that besides having an efficient solvent efflux pump, P. putida S12 and P. putida KT2440 are inherently more robust in the presence of toluene and, presumably, other organic solvents than E. coli TG1 and E. coli BL21(DE3). The absence of *cis*-*trans* isomerase (*cti*), resulting in the inability to switch from *cis*- to *trans*-fatty acid in E. coli (38), may contribute to this difference in solvent tolerance. Additionally, P. putida typically has a high NAD(P)H regeneration capacity (39, 40) which can contribute to the maintenance of proton motive force during solvent extrusion by the RND efflux pump. Further detailed investigation is required to reveal the exact basis for its intrinsic robustness.

### Identification of the novel antitoxin-toxin module SlvAT.

In P. putida S12, deletion of *srpABC* genes still resulted in higher solvent tolerance than the pTTS12-cured genotypes (Fig. 2, panel 3). This finding indicated that within pTTS12 there were other gene(s) which may play a role in solvent tolerance. Two genes of unknown function were upregulated in a transcriptome analysis of toluene-shocked P. putida, namely, RPPX_26255 and RPPX_26260, suggesting a putative role in solvent tolerance (11). Here, we confirmed this finding and demonstrated that these genes together form a novel toxin-antitoxin module (Fig. 7). SlvT exerts toxicity by degradation of NAD^+^, like other toxins of the COG5654 family, and expression of antitoxin SlvA immediately restored NAD^+^ levels. Depletion of NAD^+^ interfered with DNA replication and caused an arrest of cell division similar to another recently described COG5654-COG5642 family toxin-antitoxin pair (33). Indeed, the SlvAT toxin-antitoxin module was shown to be important for the stability of pTTS12 (Fig. 8).

Based on TADB2.0 analysis, pTTS12 encodes three TA pairs, namely, SlvAT and two identical copies of VapBC. VapBC was first identified from a virulence plasmid of *Salmonella* sp. and is known to prevent the loss of plasmid during nutrient-limiting conditions (41). A previous report showed that VapBC can stabilize/retain approximately 90% of the pUC plasmid in E. coli within 300 h of growth (42), which is similar to our result although demonstrated in a much smaller plasmid and under the control of the lac operon. Serendipitous plasmid loss due to double-strand break was reported in pSTY, which carries two identical copies of VapBC (27). Here, we observed a similar phenomenon in pTTS12 Δ*slvAT*. Hence, in the absence of SlvAT, two copies of VapBC were not sufficient to prevent the loss of pTTS12 on rich media without selection pressure and by double-strand break, indicating a major role for SlvAT.

### A putative role of toxin-antitoxin module SlvAT in solvent tolerance.

Toxin-antitoxin modules are known to be important in antibiotic persistent strains as a trigger to enter and exit the dormant state, causing the cell to become unaffected by the antibiotic (42). Among *Pseudomonas* species, several toxin-antitoxin modules are reported to be involved in survival strategies, such as stress response, biofilm formation, and antimicrobial persistence (33, 43–45). Previous transcriptomic studies reported upregulation of the *slvAT* locus as a response toward toluene addition and its expression at 10 to 30 minutes after toluene addition (11). Here, we show that SlvAT improves solvent tolerance in P. putida and E. coli strains independent of pTTS12 or the SrpABC efflux pump. We hypothesize that SlvAT plays a role as a rapid response toward toluene addition. Activation of the SlvT toxin may halt bacterial growth, and this allows physiological adaptation and adjustments to take place (e.g., expression of extrusion pumps and membrane compaction) before resuming its growth and cell division in the presence of toxic organic solvent. It is interesting to note that P. putida S12 and KT2440 both carry another COG5654-COG5642 family toxin-antitoxin pair in their chromosome (locus tag RPPX_19375-RPPX_19380 and PP_2433-PP_2434, respectively). In P. putida S12, this TA module is not being induced during solvent stress, rendering it unlikely to play a role in solvent tolerance.

### The putative regulation mechanism of toxin-antitoxin module SlvAT in P. putida S12.

Expression of the *slvAT* locus with its native promoter region seemed to exert a similar physiological effect in solvent tolerance both in E. coli and P. putida (Fig. 3 and 4). Typically, toxin-antitoxin can regulate its own expression by antitoxin binding to the promoter region (46). Unstable antitoxin is encoded upstream of the stable toxin, giving a transcriptional advantage for the production of antitoxin (47). While this study presents a role of the SlvAT module as a response to solvent stress, this toxin-antitoxin module may play a role in the response to various other stresses since pTTS12 itself encodes several modules involved in different stress response. It would be interesting to further study whether organic solvents directly induce the expression of the *slvAT* locus or intermediate signaling pathways are required. Several type II toxin-antitoxin modules are known to be regulated by proteases, such as Lon and Clp (48). These proteases degrade the antitoxin protein, promoting toxin activity, and thus upregulate the expression of the toxin-antitoxin locus. Indeed, our preliminary transcriptomic data show upregulation of specific protease-encoding loci after toluene addition. They may constitute putative regulatory proteases to the SlvAT module. Future research on the dynamics of *slvAT* locus regulation is required for revealing the details of the control mechanisms operating *in vivo*.

### Conclusions.

In summary, our experiments confirmed that the SrpABC efflux pump is the major contributor of solvent tolerance on the megaplasmid pTTS12 which can be transferred to other non-solvent-tolerant host microbes. In addition, the megaplasmid carries the novel toxin-antitoxin system SlvAT (RPPX_26255 and RPPX_26260) which promotes rapid solvent tolerance in P. putida S12 and is important for maintaining the plasmid stability of pTTS12. Chromosomal introduction of the *srpRSABC* operon genes in combination with *slvAT* confers a clear solvent tolerance phenotype in other industrial strains previously lacking this phenotype, such as P. putida KT2440, E. coli TG1, and E. coli BL21(DE3). Taken together, our findings show that both SrpABC and SlvAT constitute suitable candidate loci for exchange with various microbial hosts for increasing tolerance toward toxic compounds.

## MATERIALS AND METHODS

### Strains and culture conditions.

Strains and plasmids used in this study are listed in Table 1. All P. putida strains were grown in lysogeny broth (LB) at 30°C with 200 rpm shaking. E. coli strains were cultivated in LB at 37°C with 250 rpm shaking. For solid cultivation, 1.5% (wt/vol) agar was added to LB. M9 minimal medium was supplemented with 2 mg liter^−1^ MgSO_4_ and 0.2% of citrate as a sole carbon source (43). Toluene atmosphere growth was evaluated on solid LB in a glass plate incubated in an exicator with toluene supplied through the gas phase at 30°C. Solvent tolerance analysis was performed by growing P. putida S12 genotypes in LB starting from OD_600_ of 0.1 in Boston bottles with Mininert bottle caps. When required, gentamicin (25 mg liter^−1^), ampicillin (100 mg liter^−1^), kanamycin (50 mg liter^−1^), indole (100 g liter^−1^), potassium tellurite (6.75 to 200 mg liter^−1^), arabinose (0.8% m/v), and IPTG (2 mM) were added to the medium.

**TABLE 1:** Strains and plasmids used in this study

Strain, genotype, or plasmid	Characteristic(s)	Reference
Strain or genotype		
*P. putida*		
S12	Wild-type *P. putida* S12 (ATCC 700801), harboring megaplasmid pTTS12	3
S12-1	*P. putida* S12, harboring megaplasmid pTTS12 with Km^r^ marker	This study
S12-6/S12-10/S12-22	ΔpTTS12	This study
S12-9	ΔpTTS12, Gm^r^ *srpRSABC*::Tn7, complemented with megaplasmid pTTS12 (Tc^r^::*srpABC*)	This study
S12-C	*P. putida* ΔpTTS12 (S12-6/S12-10/S12-22), complemented with megaplasmid pTTS12	This study
KT2440	Derived from wild-type *P. putida* mt-2, ΔpWW0	46
*E. coli*		
HB101	*recA pro leu hsdR* Sm^r^	47
BL21(DE3)	*E. coli* B, F^−^ *ompT gal dcm lon hsdS_B_*(*r_B_*^−^*m_B_*^−^) λ(DE3) [*lacI lacUV5*-*T7p07 ind1 sam7 nin5*] [*malB*^+^]_K-12_(λ^S^)	29
DH5α λpir	*sup* E44, Δ*lacU*169 (Φ*lacZ*ΔM15), *recA*1, *endA*1, *hsdR*17, *thi*-1, *gyrA*96, *relA*1, λpir phage lysogen	48
TG1	*E. coli* K-12, *glnV44 thi-1* Δ*(lac-proAB)* Δ*(mcrB-hsdSM)5*(*r_K_*^−^*m_K_*^−^) F′ [*traD36 proAB*^+^ *lacI*^q^ *lacZΔM15*]	Lucigen
WM3064	*thrB1004 pro thi rpsL hsdS lacZ*ΔM15 RP4-1360 Δ(*araBAD*)567 Δ*dapA1341*::[erm pir]	William Metcalf
Plasmid		
pRK2013	RK2-Tra^+^, RK2-Mob^+^, Km^r^, *ori* ColE1	49
pEMG	Km^r^, Ap^r^, *ori* R6K, *lacZ*α MCS flanked by two I-SceI sites	37
pEMG-Δ*srpABC*	pEMG plasmid for constructing *P. putida* S12 Δ*srpABC*	This study
pEMG-Δ*slvAT*	pEMG plasmid for constructing *P. putida* S12 Δ*slvAT*	This study
pEMG-Δ*slvT*	pEMG plasmid for constructing *P. putida* S12 Δ*slvT*	This study
pSW-2	Gm^r^, *ori* RK2, *xylS*, Pm → I-SceI	37
pBG35	Km^r^, Gm^r^, *ori* R6K, pBG derived	44
pBG-srp	Km^r^, Gm^r^, *ori* R6K, pBG derived, contains *srp* operon (RPPX_27995-RPPX_27965)	This study
pBG-slv	Km^r^, Gm^r^, *ori* R6K, pBG derived, contains *slv* gene pair (RPPX_26255-RPPX_26260)	This study
pBG-srp-slv	Km^r^, Gm^r^, *ori* R6K, pBG derived, contains *slv* gene pair (RPPX_26255-RPPX_26260) and *srp* operon (RPPX_27995-RPPX_27965)	This study
pBAD18-slvT	Ap^r^, ara operon, contains *slvT* (RPPX_26260)	This study
pUK21-slvA	Km^r^, lac operon, contains *slvA* (RPPX_26255)	This study
pTnS-1	Ap^r^, *ori* R6K, TnSABC+D operon	50

### DNA and RNA methods.

All PCRs were performed using Phusion polymerase (Thermo Fisher) according to the manufacturer’s manual. Primers used in this study (Table 2) were procured from Sigma-Aldrich. PCR products were checked by gel electrophoresis on 1% (wt/vol) Tris-borate-EDTA (TBE) agarose containing 5 μg ml^−1^ ethidium bromide (110 V, 0.5× TBE running buffer). For reverse transcriptase quantitative PCR (RT-qPCR) analysis, RNA was extracted using TRIzol reagent (Invitrogen) according to the manufacturer’s manual. The obtained RNA samples were immediately reverse transcribed using the iScript cDNA synthesis kit (Bio-Rad), and cDNA may have been stored at –20°C prior to qPCR analysis. qPCR was performed using iTaq universal SYBR green supermix (Bio-Rad) on a CFX96 Touch real-time PCR detection system (Bio-Rad). The genome sequence of P. putida S12 ΔpTTS12 was analyzed using an Illumina HiSeq instrument (GenomeScan BV, The Netherlands) and assembled according to the existing complete genome sequence (GenBank accession no. CP009974 and CP009975) (12).

**TABLE 2:** Primers used in this study

Primer	Sequence (5′–3′)	Restriction site	PCR template	PCR description
TS1-srp-for	TATCTGGTACCTTGTCCTGGAAGCCGCTAATGA	KpnI	pTTS12	Construction of pEMG-*ΔsrpABC*
TS1-srp-rev	CAGCGGCGGCCGCTTTAACGCAGGAAAGCTGCGAG	NotI	pTTS12	Construction of pEMG-*ΔsrpABC*
TS2-srp-for	CCGAAGCGGCCGCCAGCGCAGTTAAGGGGATTACC	NotI	pTTS12	Construction of pEMG-*ΔsrpABC*
TS2-srp-rev	TCAGCTCTAGAGCGCAGGTAAGGCTTCACC	XbaI	pTTS12	Construction of pEMG-*ΔsrpABC*
srpO_F	TGCGAATTCGGTATCGCACATGGCATTGG	EcoRI	pTTS12	Construction of pBG-*srp*
srpO_R	TGCTCTAGAGCCTCACACCTGGTGTACC	XbaI	pTTS12	Construction of pBG-*srp*
slv_F	ATGCTTAATTAACTTTTGCTGCGGTCTACACAGG	PacI	pTTS12	Construction of pBG-*slv*
slv_R	AGCGGGAATTCCTCCAAAACCGGTTCTGAAGCC	EcoRI	pTTS12	Construction of pBG-*slv*
slvA_F	AGAGAGCTCCATAGTAAGTGCAATCCTAAAG	SacI	pTTS12	Construction of pUK21-*slvA*
slvA_R	GTCTAGACTCCAGCTCCAGATGTAG	XbaI	pTTS12	Construction of pUK21-*slvA*
slvT_F	GGTGCTCTAGAATGAAAATCATCGGAGTG	XbaI	pTTS12	Construction of pBAD18-*slvT*
slvT_R	GGAAGGAGCTCGTACGTGTAAGGCGCTAC	SacI	pTTS12	Construction of pBAD18-*slvT*
TS1_slv_F	TGCTGGAATTCCTTTTGCTGCGGTCTACACAGG	EcoRI	pTTS12	Construction of pEMG-Δ*slvAT*
TS1_slv_R	GGCAACTGATCGGTGAAAAGCACTTTGAGAGCGTCCATCAAGCC		pTTS12	Construction of pEMG-Δ*slvAT*
TS2_slv_F	GGCTTGATGGACGCTCTCAAAGTGCTTTTCACCGATCAGTTGC		pTTS12	Construction of pEMG-Δ*slvAT*
TS2_slv_R	GCCCAGGATCCCGAATGTCCATAATCCAGGCGC	KpnI	pTTS12	Construction of pEMG-Δ*slvAT*
TS1_slvT_F	GCATAGGATCCGAGAATTGTGCATAGTAAGTG	Kpn	pTTS12	Construction of pEMG-Δ*slvT*
TS1_slvT_R	GATCGTTGACCACAATATCTCCAGCTCCAGATGTAG		pTTS12	Construction of pEMG-Δ*slvT*
TS2_slvT_F	CTACATCTGGAGCTGGAGATATTGTGGTCAACGATC		pTTS12	Construction of pEMG-Δ*slvT*
TS2_slvT_R	AGGTTAAGCTTGTCTGCAGTGTCTATTCC	HindIII	pTTS12	Construction of pEMG-Δ*slvT*
eco_gyrB_F	CGATAATTTTGCCAACCACGAT		*gyrB*	qPCR, reference gene
eco_gyrB_R	GAAATTCTCCTCCCAGACCAAA		*gyrB*	qPCR, reference gene
eco_rpoB_F	AACACGAGTTCGAGAAGAAACT		*rpoB*	qPCR, reference gene
eco_rpoB_R	CGTTTAACCGCCAGATATACCT		*rpoB*	qPCR, reference gene
ppu_gyrB_F	GCTTCGACAAGATGATTTCGTC		*gyrB*	qPCR, reference gene
ppu_gyrB_R	GCAGTTTGTCGATGTTGTACTC		*gyrB*	qPCR, reference gene
ppu_rpoB_F	GACAAGGAATCGTCGAACAAAG		*rpoB*	qPCR, reference gene
ppu_rpoB_R	GAAGGTACCGTTCTCAGTCATC		*rpoB*	qPCR, reference gene
srpA_F	CTCGGAAAACTTCAGAGTTCCT		*srpA*	qPCR, target gene
srpA_R	AAAGCTTCTTGGTCTGCAAAAG		*srpA*	qPCR, target gene
srpB_F	TACATGACCAGGAAGACCAGTA		*srpB*	qPCR, target gene
srpB_R	GTGGAGGTCATTTATCCCTACG		*srpB*	qPCR, target gene
srpC_F	GCCATAAGTTGATGTTCAGCAG		*srpC*	qPCR, target gene
srpC_R	ATTCCAACGGATTTGCCAAAAA		*srpC*	qPCR, target gene

### Curing and complementation of megaplasmid pTTS12 from P. putida S12.

P. putida S12 was grown in LB to reach early-exponential phase (approximately 3 h or OD_600_ of 0.4 to 0.6). Subsequently, mitomycin C was added to the liquid LB culture to a final concentration of 10, 20, 30, 40, or 50 μg/ml. These cultures were grown for 24 h and plated on M9 minimal medium supplemented with indole to select for the absence of the megaplasmid. Loss of the megaplasmid was confirmed by a loss of other phenotypes connected with the megaplasmid, such as MIC reduction of potassium tellurite and solvent sensitivity under toluene atmosphere, as well as through genomic DNA sequencing. Complementation of megaplasmid pTTS12 was performed using biparental mating between P. putida S12-1 (pTTS12 Km^r^) and plasmid-cured genotype P. putida S12 ΔpTTS12 (Gm^r^:Tn7) and followed by selection on LB agar supplemented with kanamycin and gentamicin.

### Plasmid cloning.

Deletion of *srpABC*, *slvT*, and *slvAT* genes was performed using homologous recombination between free-ended DNA sequences that are generated by cleavage at unique I-SceI sites (37). Two homologous recombination sites were chosen downstream (TS-1) and upstream (TS-2) of the target genes. TS-1 and TS-2 fragments were obtained by performing PCR using primers listed in Table S1. Constructs were verified by DNA sequencing. Mating was performed as described by Wynands and colleagues (27). Deletion of *srpABC*, *slvT*, and *slvAT* was verified by PCR and Sanger sequencing (Macrogen B.V., Amsterdam, The Netherlands).

Introduction of the complete *srp* operon (*srpRSABC*) and *slvAT* was accomplished using the mini-Tn7 delivery vector backbone of pBG35 developed by Zobel and colleagues (44). The DNA fragments were obtained by PCR using primer pairs listed in Table 2 and ligated into the pBG35 plasmid at PacI and XbaI restriction sites. This construct generated a Tn7 transposon segment in pBG35 containing a gentamicin resistance marker and *srp* operon with Tn7 recognition sites flanking on the 5′ and 3′ sides of the segment. Restriction analysis followed by DNA sequencing (Macrogen, The Netherlands) were performed to confirm the correct pBG-srp, pBG-slv, and pBG-srp-slv construct. The resulting construct was cloned in E. coli WM3064 and introduced into P. putida or E. coli strains with the help of E. coli WM3064 pTnS-1. Integration of the construct into the Tn7 transposon segment was confirmed by gentamicin resistance, PCR, and the ability of the resulting transformants to withstand and grow under toluene atmosphere conditions.

### Toxin-antitoxin assay.

Bacterial growth during the toxin-antitoxin assay was observed in LB medium supplemented with 100 mg liter^−1^ ampicillin and 50 mg liter^−1^ kanamycin. Starting cultures were inoculated from a 1:100 dilution of overnight culture (OD_600_, 0.1) into a microtiter plate (96 well), and bacterial growth was measured using a Tecan Spark 10M instrument. To induce toxin and antitoxin, a total concentration of 0.8% (m/vol) arabinose and 2 mM IPTG were added to the culture, respectively. Cell morphology was observed using a light microscope (Zeiss Axiolab 5) at a magnification of ×100. A final concentration of 2.5× SYBR green I (10,000× stock; New England BioLabs) was applied to visualize DNA, followed by two times washing with 1× phosphate-buffered saline (PBS), and analyzed using a Guava easyCyte single sample flow cytometer (Millipore). At indicated time points, NAD^+^ levels were measured using a NAD/NADH-Glo assay kit (Promega) according to the manufacturer’s manual. The percentage of the NAD^+^ level was calculated by dividing the measured luminescence of tested strains with that of the control strains at the same time points. RPPX_26255 and RPPX_26260 were modeled using the I-TASSER server (35) and visualized using PyMol (version 2.3.1). Phylogenetic trees of toxin-antitoxin module derived from the COG5654-COG5642 family were constructed using MEGA (version 10.0.5) as a maximum likelihood tree with 100 bootstraps and visualized using the iTOL Web server (https://itol.embl.de) (45).

### Data availability.

The sequence data for wild-type *P. putida* S12 and plasmid-cured genotypes *P. putida* S12 ΔpTTS12 have been submitted to the SRA database under accession number PRJNA602416.

## Supplementary Material

Supplemental file 1
