# Supplementary material for: Novel Toxin-Antitoxin Module SlvT-SlvA Regulates Megaplasmid Stability and Incites Solvent Tolerance in Pseudomonas putida S12
Source: Appl Environ Microbiol. 2020 Jun 17;86(13):e00686-20. doi: 10.1128/AEM.00686-20 (PMC7301842; doi:10.1128/AEM.00686-20)
Supplement: Supplemental file 1 [file AEM.00686-20-s0001.pdf]

Supplemental Material

A novel toxin-antitoxin module SlvT–SlvA regulates megaplasmid stability and incites solvent tolerance in *Pseudomonas putida* S12

Hadiastri Kusumawardhani, David van Dijk, Rohola Hosseini, Johannes H. de Winde*

Institute of Biology Leiden, Leiden University, The Netherlands

*Correspondence: j.h.de.winde@biology.leidenuniv.nl

Table S1. Expression of *srpABC* genes in *P. putida* and *E. coli* strains in basal level and in the presence of 0.10 % v/v toluene with *gyrB* and *rpoB* as reference genes

Genes	Strains	Basal expression	Toluene induced expression	Fold change
srpA	*P. putida* S12	1.69 ± 0.31	4.08 ± 0.79	2.44 ± 0.50
	P. putida S12-6.1	2.27 ± 0.36	4.56 ± 1.08	1.99 ± 0.28
	P. putida KT2440-srp	2.84 ± 0.92	5.60 ± 1.79	1.99 ± 0.28
	E. coli BL21(DE3)-srp	3.30 ± 0.43	6.60 ± 2.06	1.98 ± 0.45
	E. coli TG1-srp	5.28 ± 1.76	11.06 ± 4.10	2.10 ± 0.36
srpB	*P. putida* S12	1.92 ± 0.36	3.83 ± 0.76	2.02 ± 0.42
	P. putida S12-6.1	2.57 ± 0.33	5.49 ± 1.13	2.15 ± 0.46
	P. putida KT2440-srp	3.51 ± 1.22	6.59 ± 1.87	1.91 ± 0.20
	E. coli BL21(DE3)-srp	3.69 ± 0.58	7.52 ± 2.26	2.01 ± 0.36
	E. coli TG1-srp	5.35 ± 1.97	11.52 ± 4.84	2.21 ± 0.75
srpC	*P. putida* S12	2.01 ± 0.28	3.96 ± 0.75	1.97 ± 0.29
	P. putida S12-6.1	2.32 ± 0.27	4.45 ± 0.99	1.91 ± 0.35
	P. putida KT2440-srp	2.07 ± 0.68	5.13 ± 1.55	2.50 ± 0.22
	E. coli BL21(DE3)-srp	3.41 ± 0.52	6.70 ± 1.67	1.95 ± 0.25
	E. coli TG1-srp	4.18 ± 1.40	9.78 ± 5.23	2.32 ± 0.92

Table S2. Codon adaptation index of *srp* operon in *E. coli* and *P. putida* reference strains

Genes	Length	CAI	%G+C	Nc	Species	Strain
srpA	1170	0.598	56.5	57.2	*E.coli*	K12
	1170	0.6	56.5	57.2	*E.coli*	B
	1170	0.742	56.5	57.2	*E.coli*	N/A
	1170	0.442	56.5	57.2	*P.putida*	F1
	1170	0.468	56.5	57.2	*P.putida*	N/A
	1170	0.372	56.5	57.2	*P.putida*	GB1
srpB	3150	0.662	56.5	46.1	*E.coli*	K12
	3150	0.67	56.5	46.1	*E.coli*	B
	3150	0.749	56.5	46.1	*E.coli*	N/A
	3150	0.565	56.5	46.1	*P.putida*	F1
	3150	0.587	56.5	46.1	*P.putida*	N/A
	3150	0.502	56.5	46.1	*P.putida*	GB1
srpC	1413	0.62	54.2	51.3	*E.coli*	K12
	1413	0.628	54.2	51.3	*E.coli*	B
	1413	0.735	54.2	51.3	*E.coli*	N/A
	1413	0.456	54.2	51.3	*P.putida*	F1
	1413	0.483	54.2	51.3	*P.putida*	N/A
	1413	0.394	54.2	51.3	*P.putida*	GB1
srpR	642	0.604	54.5	59.8	*E.coli*	K12
	642	0.622	54.5	59.8	*E.coli*	B
	642	0.724	54.5	59.8	*E.coli*	N/A
	642	0.464	54.5	59.8	*P.putida*	F1
	642	0.491	54.5	59.8	*P.putida*	N/A
	642	0.399	54.5	59.8	*P.putida*	GB1
srpS	780	0.636	55.4	55.5	*E.coli*	K12
	780	0.638	55.4	55.5	*E.coli*	B
	780	0.737	55.4	55.5	*E.coli*	N/A
	780	0.47	55.4	55.5	*P.putida*	F1
	780	0.496	55.4	55.5	*P.putida*	N/A
	780	0.399	55.4	55.5	*P.putida*	GB1

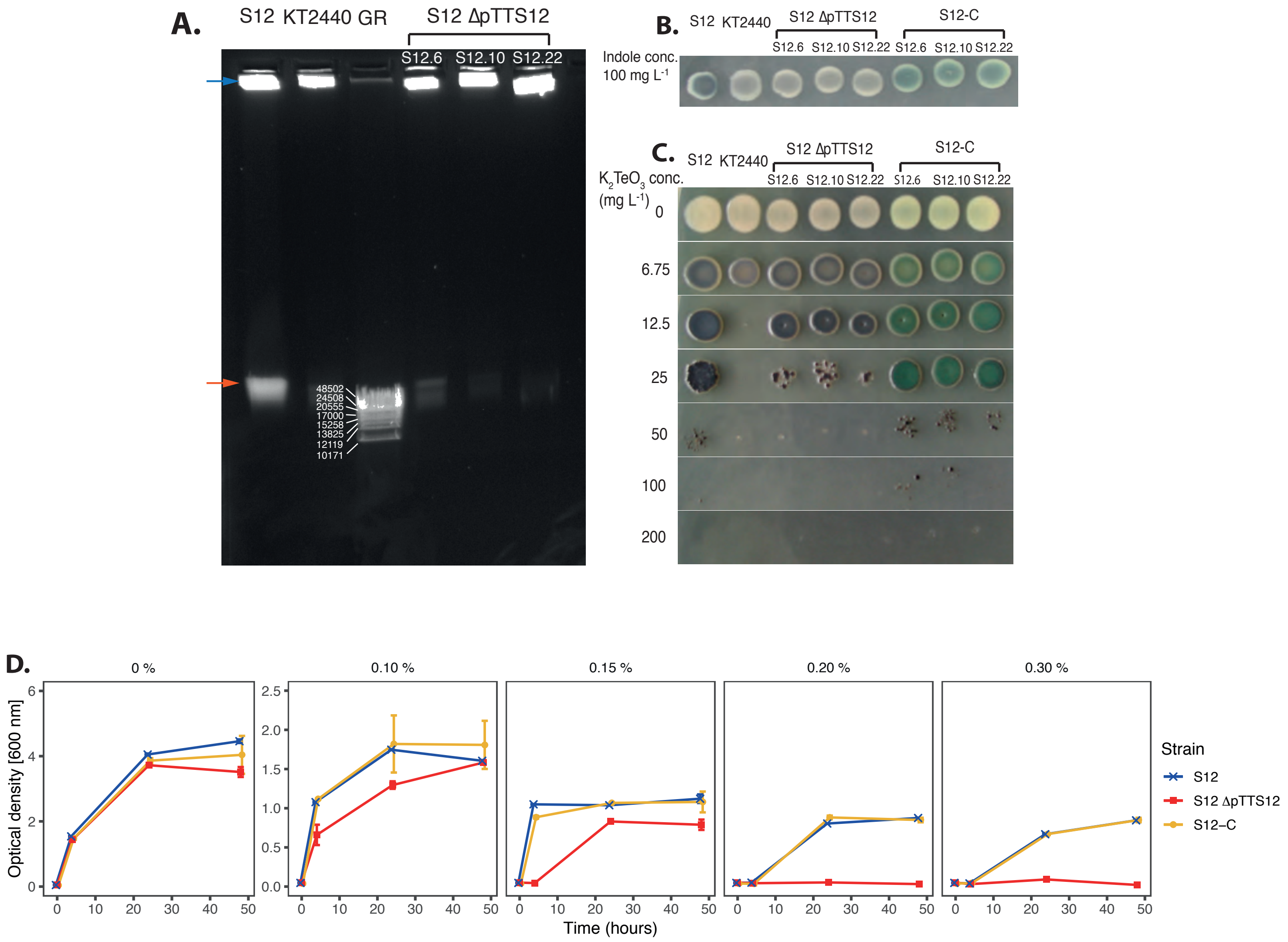

Figure S1. Removal and complementation of the megaplasmid pTTS12 from *P. putida* S12.

A. The loss of the megaplasmid band in megaplasmid-cured *P. putida* S12 proven by electrophoresis of agarose embedded genomic DNA. Megaplasmid band (orange arrow) was visible in the positive control *P. putida* S12 and absent in negative control *P. putida* KT2440 and Mitomycin C treated strains (strain S12-6, S12-10, and S12-22). Blue arrow indicates bacterial chromosome.

B. Activity of styrene monooxygenase (SMO) and styrene oxide isomerase (SOI) for indigo formation from indole in *P. putida* strains. Enzyme activity was lost in the megaplasmid-cured strains S12 ΔpTTS12 (white colonies) and restored with the complementation of megaplasmid in the strains S12-C (blue colonies). Indole (100 mg L^{-1}) was supplemented in M9 minimum media.

C. K_2TeO_3 resistance of *P. putida* strains on lysogeny broth (LB) agar. Tellurite resistance was reduced in the megaplasmid-cured strains S12 ΔpTTS12 (MIC 50 mg L^{-1}) and restored with the complementation of meg-aplasmid in the strains S12-C (MIC 200 mg L^{-1}).

D. Solvent tolerance analysis was performed on *P. putida* S12, *P. putida* S12 ΔpTTS12, and *P. putida* S12-C growing in liquid LB media with 0, 0.10, 0.15, 0.20 and 0.30 % v/v toluene. The removal of the megaplasmid pTTS12 clearly caused a significant reduction in the solvent tolerance of *P. putida* S12 ΔpTTS12. Complemen-tation of pTTS12 restores the solvent tolerance trait in *P. putida* S12-C. This figure displays the mean of three independent replicates and error bars indicate standard deviation. The range of y-axis is different in the first panel (0 - 6) than the rest of the panels (0 - 2.5).

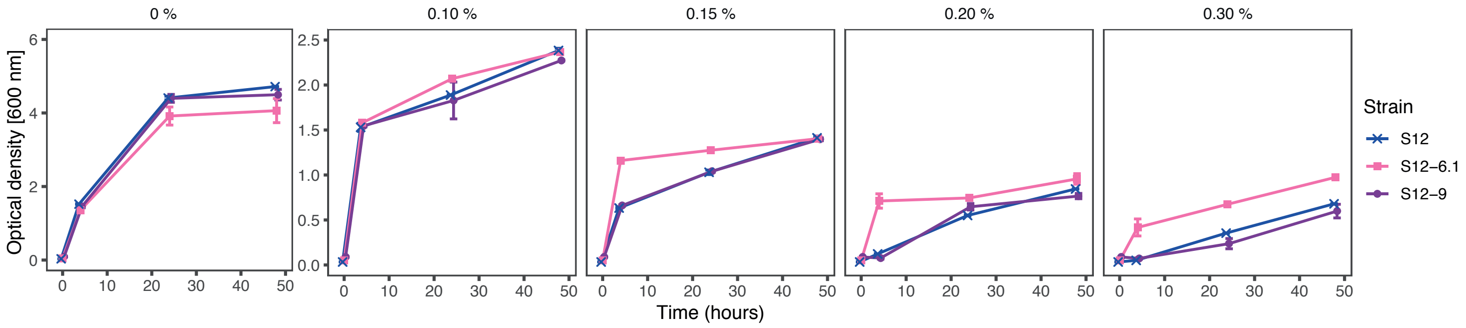

Figure S2. Metabolic burden of megaplasmid pTTS12 during growth in the presence of organic solvent.
Solvent tolerance was compared between *P. putida* S12, *P. putida* S12-6.1 (S12-6 *srp*::attn7), and *P. putida* S12-9 (S12-6 *srp*::attn7, pTTS12 tet::*srp*) in liquid LB media with 0, 0.10, 0.15, and 0.20 % v/v toluene. This figure displays the mean of three independent replicates and error bars indicate standard deviation. The range of y-axis is different in the first panel (0 - 6) than the rest of the panels (0 - 2.5).

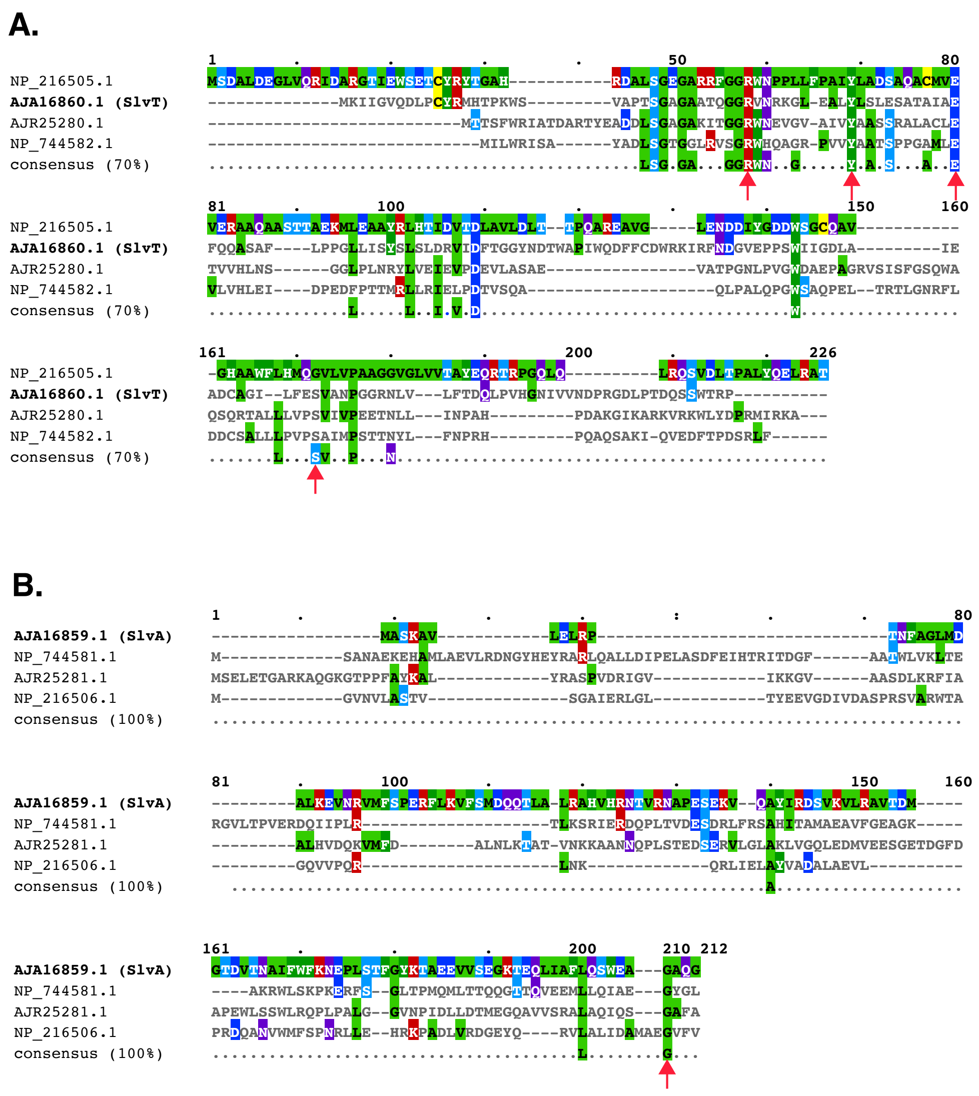

Figure S3. Multiple alignment of SlvT and SlvA with characterized toxin-antitoxin of COG5654-COG5642 family

Sequence similarity of the COG5654 toxin SlvT (A) from *P. putida* S12 and COG5642 antitoxin SlvA (B) with several characterized COG5654-COG5642 family toxin-antitoxin protein. Putative active site residues showed >70% similarities and are indicated by red arrows.
